# Multidisciplinary Collaboration in Combatting Antimicrobial Resistance: Insights and Outcomes From the National Alliance of Medical Professionals on Antimicrobial Resistance (NAMP-AMR) Initiative

**DOI:** 10.7759/cureus.68932

**Published:** 2024-09-08

**Authors:** Venkatesh Karthikeyan, Narender Saini, Ramyaramalakshmi Nadarajan, Meenakshi Gopimohan, Parvati Roy

**Affiliations:** 1 Community and Family Medicine, All India Institute of Medical Sciences, Patna, Patna, IND; 2 Standing Committee for Antimicrobial Resistance, Indian Medical Association, Delhi, IND

**Keywords:** antibiotics, antimicrobial resistance, antimicrobial resistance (amr), namp amr, nap amr, national action plan on antibiotic resistance, national alliance of medical professionals on antimicrobial resistance

## Abstract

Antimicrobial resistance (AMR) poses a critical global health challenge, requiring a coordinated and vigorous response. Despite numerous global and national efforts, a unified multidisciplinary approach has been missing. The National Alliance of Medical Professionals on Antimicrobial Resistance (NAMP-AMR), led by the Indian Medical Association in collaboration with key stakeholders such as NITI Aayog and the Ministry of Health, seeks to address this gap. This alliance unites 52 medical specialty organizations and associations to enhance efforts across various sectors, developing comprehensive strategies for awareness, surveillance, infection control, and optimization of antimicrobial use. The foundational meeting of NAMP-AMR on July 7, 2024, established a collaborative roadmap, promising to bolster India’s efforts against AMR and support the forthcoming National Action Plan on AMR 2.0. The meeting concentrated on six key areas: improving AMR awareness and advocacy; strengthening laboratory capacities and surveillance; enhancing infection prevention and control (IPC); optimizing antimicrobial use through stricter regulations and stewardship programs; advancing AMR research and innovation; and fostering strong national and international collaborations. This initiative marks a significant advancement in combating AMR and positions India as a leader in global health efforts against this critical issue.

## Introduction

Antimicrobial resistance (AMR) is a significant global health threat, resulting in increased mortality and morbidity. The WHO has declared AMR a “silent pandemic,” underscoring the urgency of addressing this issue [1]. AMR poses a worldwide threat, with drug-resistant diseases claiming the lives of 4.95 million individuals in 2019, including 1.27 million deaths directly related to AMR [2]. Notably, children under the age of five accounted for one in five of these fatalities [3]. In India, the impact of AMR is profound, with 1,042,500 deaths associated with AMR and 297,000 deaths attributable to AMR in 2019 [4]. Among 204 nations, India ranks 145th in terms of age-standardized death rates per 100,000 people linked to AMR [4].

Recognizing this growing burden, the WHO has made AMR a global priority. Initiatives to combat AMR have included the launch of the Global Action Plan on AMR by the WHO in 2015, setting the stage for a coordinated international response [5]. In the same year, the Global Antimicrobial Resistance and Use Surveillance System (GLASS) was launched to fill knowledge gaps and inform strategies at all levels [6]. In 2016, AMR was featured as a critical health topic, and the multisectoral annual “Tracking AMR Country Self-Assessment Survey" (TrACSS) was launched [7]. To steer research and development of new antimicrobials, diagnostics, and vaccines, and to inform public health strategies, the WHO created the inaugural WHO bacterial priority pathogens list in 2017 [8]. To coordinate the One Health global response to AMR, the WHO has collaborated with the Food and Agriculture Organization, the United Nations Environment Programme (UNEP), and the World Organisation for Animal Health (WOAH), collectively known as the Quadripartite. A joint secretariat hosted by WHO facilitates multi-stakeholder engagement, supporting the Global Leaders Group on AMR, which commenced its activities in November 2020 [9]. As of November 2023, 178 countries had formulated national action plans for AMR that are in alignment with the Global Action Plan [10]. Most recently, in 2024, a resolution on AMR was adopted at the World Health Assembly, underscoring the urgency of the issue [11].

In India, the battle against AMR is spearheaded by the Ministry of Health and Family Welfare (MOHFW). A significant milestone was the launch of the “National Programme on AMR Containment” in 2013 by the National Centre for Disease Control (NCDC), marking a pivotal step in the nation's response [12]. The National Action Plan on Antimicrobial Resistance, launched in 2017, adopts a One Health approach and includes various stakeholder ministries and departments [13]. This was further reinforced by the Delhi Declaration on AMR, an inter-ministerial consensus where ministers pledged support for AMR containment [14]. Following the national framework, several states have initiated their respective state action plans, demonstrating regional commitment to combating AMR [15]. Looking forward, the development of the National Action Plan 2.0 aims to expand upon prior efforts and introduce innovative strategies [16].

Despite numerous global and national initiatives aimed at curbing AMR, the challenge continues to escalate. These global and national efforts often lack coordination and the comprehensive engagement of all stakeholders necessary to effect substantial change. Hence, to address this critical gap, the Indian Medical Association, in collaboration with NITI Aayog, MOHFW, Directorate General of Health Services, World Health Association - India, NCDC, and 52 medical associations/organizations, has formed the National Alliance of Medical Professionals on Antimicrobial Resistance (NAMP-AMR). NAMP-AMR seeks to bridge these gaps by uniting medical professionals across disciplines and specialties, fostering a unified approach that amplifies impact, drives cohesive policy changes, and enhances educational outreach on a scale not previously achieved. This initiative is poised to catalyze significant advancements in AMR through its integrated, multi-stakeholder strategy.

The inaugural consultation meeting of the NAMP-AMR convened on July 7, 2024, at the Indian Medical Association Headquarters in Delhi. This landmark gathering brought together a distinguished roster of participants, including Dr. VK Paul (NITI Aayog), Dr. R V Asokan (National President of IMA), Dr. Atul Goel (Director General of Health Services), Ms. Payden (Country Deputy Head of WHO India), Dr. Lata Kapoor (National Center for Disease Control), Dr. Atul Kocchar (CEO of the National Accreditation Board of Hospitals), and Dr. Purushottam Giri (Secretary General, Indian Association of Preventive and Social Medicine). Additionally, presidents, secretaries, and representatives from 52 medical specialties and organizations attended. The meeting was structured around three pivotal objectives: first, to disseminate information regarding ongoing AMR initiatives; second, to develop a consensus on addressing AMR as a shared priority; and third, to forge a collaborative roadmap for the future activities of NAMP-AMR, setting the stage for a unified and effective response to this global health threat.

## Materials and methods

Study design

The study employed a qualitative design, leveraging a series of structured discussions, presentations, and collaborative planning sessions during the first NAMP-AMR consultation meeting held on July 7, 2024, at the Indian Medical Association Headquarters in Delhi, India.

Study participants

**Figure 1:**
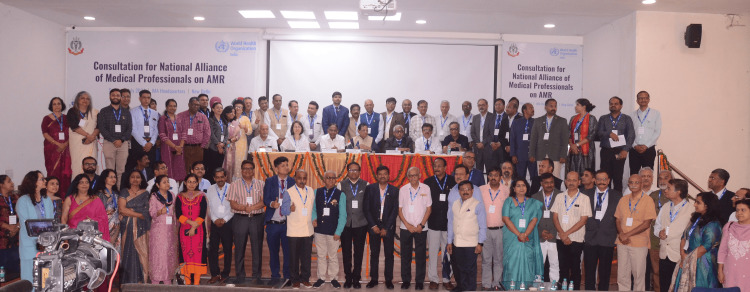
The inaugural consultation meeting of the National Alliance of Medical Professionals on Antimicrobial Resistance, held on July 7, 2024, at the Indian Medical Association headquarters in Delhi, India

Participants included representatives from 52 medical specialty associations/organizations, government officials from health-related agencies, including representatives from NITI Aayog, MOHFW, the Directorate General of Health Services, the National Center for Disease Control, the Indian Medical Association, experts from the WHO, and other stakeholders in the healthcare industry (Figure 1).

The list of participant associations/organizations is listed in Table 1.

**Table 1:** Members of the National Alliance of Medical Professionals on Antimicrobial Resistance

Members of the National Alliance of Medical Professionals on Antimicrobial Resistance
NITI Aayog (National Institution for Transforming India)
Ministry of Health and Family Welfare, Government of India
Directorate General of Health Services (DGHS)
Indian Medical Association (IMA)
WHO India
National Centre for Disease Control (NCDC)
National Accreditation Board for Hospitals and Healthcare Providers (NABH)
All India Rhinology Society (AIRS)
Association of Clinical Biochemists of India (ACBI)
Association of Microbiologists of India (AMI)
Association of Oral and Maxillofacial Surgeons of India (AOMSI)
Association of Radiation Oncologists of India (AROI)
Association of Spine Surgeons of India (ASSI)
Association of Surgeons of India (ASI)
Clinical Infectious Disease Society (CIDS)
Consortium of Accredited Healthcare Organization (CAHO)
Delhi Society for Promotion of Rational Use of Drug (DSPRUD)
Epidemiology Foundation of India (EFI)
Federation of Obstetric and Gynaecological Societies of India (FOGSI)
Geriatric Society of India (GSI)
Global Health Advocacy Incubator (GHAI)
Hospital Infection Society – India (HISI)
Indian Academy of Neurology (IAN)
Indian Academy of Otorhinolaryngology Head and Neck Surgery (IAOHNS)
Indian Academy of Pediatrics (IAP)
Indian Association of Bronchology (IAB)
Indian Association of Cardiovascular and Thoracic Surgeons (IACTS)
Indian Association of Dermatologists, Venereologists, and Leprologists (IADVL)
Indian Association of Interventional Cardiac Surgeons (IAICS)
Indian Association of Medical Microbiologists (IAMM)
Indian Association of Occupational Health (IAOH)
Indian Association of Preventive and Social Medicine (IAPSM)
Indian Association of Sports Medicine (NASM)
Indian Dental Association (IDA)
Indian Medical Association – Junior Doctors’ Network (IMA JDN)
Indian Medical Association - Medical Students' Network (IMA MSN)
Indian Public Health Association (IPHA)
Indian Radiological and Imaging Association (IRIA)
Indian Society for Trauma and Acute Care (ISTAC)
Indian Society of Clinical Research (ISCR)
Indian Society of Medical and Paediatric Oncology (ISMPO)
Indian Society of Nephrology (ISN)
Indian Society of Oncology (ISO)
Indian Society of Organ Transplantation (ISOT)
Indian Society of Periodontology (ISP)
Indian Society of Reconstructive Microsurgery (ISRM)
Indian Veterinary Association (IVA)
National Neonatal forum (NNF)
Pediatric Infectious Diseases Academy (PIDA)
Public Health Foundation of India (PHFI)
ReAct Asia Pacific
Society for Emergency Medicine India (SEMI)
The Indian College of Emergency Medicine (ICEM)
Urological Society of India (USI)
Vascular Society of India (VSI)

Data collection

**Figure 2:**
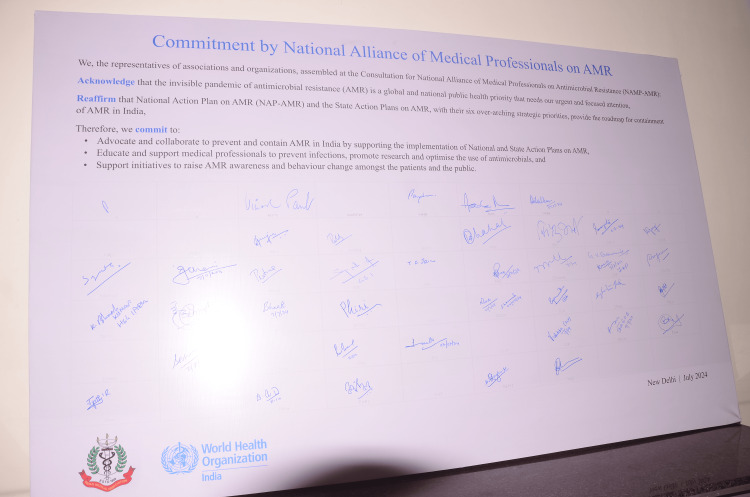
Commitment board signed by members of the National Alliance of Medical Professionals on Antimicrobial Resistance

The data collection for the NAMP-AMR study was meticulously structured to encompass a broad spectrum of inputs from a diverse group of stakeholders engaged in AMR mitigation. The approach included direct consultations, during which stakeholders discussed current strategies and challenges; e-poster presentations by member associations, detailing their AMR initiatives and future plans; and the use of a “commitment board” for associations to record their pledges toward AMR efforts (Figure 2).

Additionally, collaborative planning sessions were conducted to facilitate detailed discussions on joint AMR actions, enhancing the granularity of the data collected. Feedback forms were distributed at the conclusion of the meeting to assess its effectiveness and gather further qualitative inputs. This multifaceted data collection strategy ensured a comprehensive aggregation of insights, reflecting the varied perspectives and strategies from across the healthcare sector, vital for crafting an informed and cohesive response to AMR.

Structured themes

The consultation focused on six strategic themes, each targeting a different aspect of AMR mitigation. The first theme, Awareness, Understanding, and Advocacy, aimed to enhance public and professional knowledge of AMR through education and advocacy efforts. The second theme, Strengthening Laboratories and AMR Surveillance, concentrated on improving laboratory capacities to ensure better AMR surveillance and data accuracy. Infection Prevention and Control (IPC) was the third theme, focusing on implementing robust IPC measures in healthcare settings to reduce infection rates and prevent the spread of resistant bacteria. The fourth theme, Optimizing Antimicrobial Use, addressed regulatory and stewardship measures to promote the judicious use of antimicrobials. The fifth theme, Research and Innovation, emphasized the need for ongoing research to develop new diagnostic tools and therapeutic options, informing treatment and prevention strategies. Lastly, Collaborations highlighted the importance of national and international partnerships to foster a unified response to AMR.

Data analysis

Qualitative data from discussions and presentations were analyzed thematically according to the predefined themes to identify key strategies, commitments, and consensus points relevant to advancing the AMR agenda. Transcripts and documented outputs were coded line by line to identify recurring themes and patterns. Data coded under each theme were synthesized to construct a comprehensive view of the discussions, highlighting consensus and varying viewpoints. Preliminary findings were validated through follow-up discussions with key participants to ensure accuracy and representativeness of the interpreted data.

## Results

The study successfully engaged a broad spectrum of 52 medical specialty associations/organizations along with governmental and non-governmental stakeholders. The diversity of the participants enriched the data, providing comprehensive insights across multiple dimensions of AMR. The theme-specific findings/recommendations are as follows:

For Theme 1: Awareness, Understanding, and Advocacy, the recommendations include forming an awareness network by grouping professional associations, paraclinical staff, ground-level workers, and community members. It suggests promoting World AMR Awareness Week and utilizing celebrities and influencers for campaigns through social media, cinemas, pamphlets, hoardings, street plays, and buses. Developing state action policies with periodic reviews, integrating AMR education into academic curricula, and engaging local health authorities for funding and resources are also recommended. Additionally, promoting a One Health approach, creating scientific materials, and developing the AMAAR App for general practitioners are key points.

For Theme 2: Strengthening Laboratories and AMR Surveillance, the study recommends augmenting AMR surveillance in key laboratories across human, environmental, and veterinary health sectors, and developing guidelines for sample collection and testing quality assurance. Establishing guidelines for appropriate antibiotic use, identifying specialized laboratories, and implementing External Quality Assurance surveillance are important. The theme also highlights the need for societal-level surveillance, a hotline for antimicrobial-related advice, and a portal for real-time data reporting.

Theme 3: Infection Prevention and Control focuses on declaring IPC a key priority, implementing measures to reduce hospital-acquired infections (HAI), and training physicians on IPC. It suggests incorporating IPC topics into publications, workshops, and community awareness programs, and integrating IPC into educational curricula. Developing basic and expert IPC guidelines, establishing HAI protocols, and creating an information system for managing outbreaks are also recommended.

For Theme 4: Optimizing Antimicrobial Use, the study emphasizes recognizing delays in regulating Schedule H drugs, improving awareness among professionals and pharmacists, and enforcing strategies for prescription audits. It advocates for educating the public, enhancing OTC sales surveillance, promoting restricted drug use, and ensuring adherence to antimicrobial use principles. The theme also supports mandatory antimicrobial stewardship programs in hospitals.

Theme 5: Research and Innovation includes conducting epidemiological research, Knowledge, Attitudes, and Practices (KAP) studies, and mandating biannual antibiogram production. It emphasizes research funding for country-specific AMR data, developing vaccines, and facilitating academia-industry collaborations for effective AMR mitigation.

Finally, Theme 6: Collaborations recommends engaging in knowledge sharing with international counterparts, adopting non-antimicrobial management strategies, and organizing AMR sessions at state-level conferences. It supports establishing AMR cells in all states, creating technical groups for lagging states, and integrating AMR strategies with national surveillance programs. Engaging students and communities, monitoring best practices, and extending the alliance to relevant departments for a quadripartite approach are also highlighted.

Verification and validation of data

The themes and data points were repeatedly cross-verified with participants to ensure the accuracy and reliability of the information gathered. The synthesis of the data was shared with all contributors for final validation, ensuring that the results accurately reflected the collective inputs and consensus reached during the meeting.

The video recording of the entire session is available online in two parts (Video 1, Video 2).

**Video 1:** Video recording of the first consultation meeting of the National Alliance of Medical Professionals on Antimicrobial Resistance – Part 1

**Video 2:** Video recording of the first consultation meeting of the National Alliance of Medical Professionals on Antimicrobial Resistance – Part 2

## Discussion

The inaugural meeting of the NAMP-AMR highlighted several critical aspects of AMR management and underscored the urgent need for cohesive action. The discussions during the meeting reflected a collective acknowledgment of the severe threat posed by AMR and the complexities involved in its containment and management. The engagement of diverse medical specialties and high-level stakeholders, including governmental bodies, provided a robust platform for interdisciplinary dialogue and consensus-building.

The meeting successfully established a framework for ongoing collaboration and outlined actionable strategies for member associations to adopt. Key recommendations include allocating specific sessions for AMR discussions at every national and state conference to ensure continuous focus on the latest developments and research. Each association should designate an AMR Coordinator to oversee the implementation of AMR initiatives and maintain continuity. The AMR Pledge should be promoted among members to foster a culture of responsible antimicrobial use and stewardship. AMR and IPC should be officially recognized as primary focus areas, enhancing visibility and commitment across member websites. Active participation in World Antimicrobial Resistance Awareness Week is encouraged to raise public and professional awareness about AMR challenges and advancements. Annual publication of scientific research on AMR is recommended to contribute to global knowledge and evidence-based practices. Capacity-building programs should be implemented to enhance members' expertise in effective AMR management. Regular review meetings are essential for assessing progress, sharing best practices, and refining strategies within the NAMP-AMR framework.

These actions are designed not only to enhance the capabilities of individual associations but also to foster a strong, integrated network that can effectively address the multifaceted challenges of AMR. The discussion underscores the potential of NAMP-AMR to serve as an anchor for the National Action Plan on AMR 2.0, as emphasized by Dr. VK Paul from NITI Aayog. This strategic alignment with national policies enhances the initiative’s capacity to influence AMR outcomes significantly.

## Conclusions

The NAMP-AMR emerges as a critical element supporting the ongoing efforts to tackle the pervasive issue of AMR within India. This initiative, characterized by its broad and inclusive approach, aims to mobilize and unify the medical community, leveraging collective expertise and resources to address this global health crisis. By fostering collaboration across various medical specialties and organizations, NAMP-AMR not only enriches the dialogue around AMR but also enhances the implementation of effective strategies to mitigate its impact. This robust collaborative framework offers significant promise to transform the AMR landscape, establishing a model for integrated health governance that can be mirrored worldwide.
